# Suxiao Jiuxin Pill alleviates myocardial ischemia–reperfusion injury through the ALKBH5/GSK3β/mTOR pathway

**DOI:** 10.1186/s13020-023-00736-6

**Published:** 2023-03-23

**Authors:** Yiping Li, Ruixia Lu, Zhenchao Niu, Dan Wang, Xiaolong Wang

**Affiliations:** 1 Cardiovascular Department of Traditional Chinese Medicine, Shuguang Hospital, Affiliated to Shanghai University of Traditional Chinese Medicine, Shanghai, China; 2 Cardiovascular Research Institute of Traditional Chinese Medicine, Shuguang Hospital Affiliated to Shanghai University of Traditional Chinese Medicine, Shanghai, China

**Keywords:** Suxiao Jiuxin pill, Autophagy, Myocardial ischemia–reperfusion injury, ALKBH5/GSK3β/mTOR pathway

## Abstract

**Background:**

Many studies have shown effective protection from myocardial ischemia–reperfusion injury (MIRI) in animal models, but few, if any, treatments have yielded a substantial reduction in clinical. Several studies showed significant therapeutic effects for the Chinese patent medicine Suxiao Jiuxin Pill (SJP) in MIRI, although the specific molecular mechanisms remain undefined. Recently, increasing evidence indicates an important role for m6A modification in autophagy regulation in MIRI, and SJP has not been investigated in this regard.

**Methods:**

In vivo experiments were performed in a Wistar rat MIRI model. In vitro assays were conducted in hypoxia/reoxygenation (H/R)-treated H9c2 cells. H9c2 cells with ALKBH5 and GSK3β silencing were constructed by lentivirus transfection. TUNEL and Annexin V/PI assays were carried out for apoptosis detection. Then, m6A modification was detected with the EpiQuik m6A RNA methylation quantification kit, and GFP-RFP-LC3B was used to observe dynamic changes in autophagy. The autophagosome structure was assessed by Transmission electron microscopy. qPCR and immunoblot were performed for mRNA and protein analyses, receptively.

**Results:**

SJP significantly mitigated MIRI in rats, reducing infarct size and myocardial apoptosis, and improving left ventricular function. In addition, SJP inhibited autophagy through the GSK3β/mTOR pathway in MIRI rats. In cultured H9c2 cells, SJP significantly inhibited H/R- related apoptosis and autophagic activity through the GSK3β/mTOR pathway. Additionally, SJP enhanced ALKBH5 expression in H/R cardiomyocytes, which is important in impaired m6A modification. Interestingly, ALKBH5 knockdown enhanced autophagy and apoptosis in H/R-induced cells, whereas SJP reversed these effects. Further experiments showed that autophagic activity and apoptosis enhanced by ALKBH5 deficiency are GSK3β/mTOR pathway dependent in H/R-treated H9c2 cells. After SJP administration the above effects were alleviated, suggesting SJP inhibited autophagy through the ALKBH5/GSK3β/mTOR pathway in H/R-induced cardiomyocytes. These effects of SJP were common to its two main constituents, including tetra-methylpyrazine (TMP) and borneol (BOR).

**Conclusion:**

SJP improves MIRI in rats and alleviates autophagy and apoptosis in H9c2 cells through the ALKBH5/GSK3β/mTOR pathway, thanks to its two major constituents TMP and BOR.

**Supplementary Information:**

The online version contains supplementary material available at 10.1186/s13020-023-00736-6.

## Introduction

Approximately 17.3 million individuals die from acute myocardial infarction (AMI) worldwide each year, which is estimated to exceed 23.6 million by 2030 [1]. The best intervention for AMI is early myocardial reperfusion through revascularization. However, myocardial ischemia/reperfusion injury (MIRI) worsens patient outcome after revascularization, increasing mortality and readmission rate in the later period [2]. Previous studies have demonstrated that timely opening of infarct-related arteries through revascularization only reduces infarct size in the dangerous myocardial area (AAR) by 50%, and about half of the remaining AAR myocardial infarctions are caused by fatal but preventable MIRI [3]. Although this damage response is quite significant, no efficient treatments targeting MIRI are available [4].

Cardiomyocyte stress and reperfusion may lead to abnormal mitochondrial function, oxidative stress, and inflammation that lead to autophagy [5]. The event is controlled by number epigenetic modifications including N6 methyladenosine. The N6 methyladenosine (m6A) represents an important methylation modification of RNAs that was identified five decades ago. m6A controls alternative splicing, protein synthesis, mRNA stability, and the expression of target genes [6]. Moreover, m6A modification regulates autophagy by impacting the mRNA processing of autophagy-associated genes, suggesting an important regulatory role for this posttranscriptional modification in autophagy [7, 8], which provides a new perspective to guide the treatment of cardiovascular disease (CVD).

Numerous experimental studies have shown effective protection from MIRI in animal models, but translation into clinical practice has been less successful. The clinical application of natural products or traditional Chinese medicine (TCM) compounds may open a new avenue for MIRI therapy. Suxiao jiuxin pill (SJP) is composed of Rhizome *Ligusticum Chuanxiong* (Chuan xiong, *Ligusticum chuanxiong Hort.*) and *Dryobalanops aromatica* C.F.Gaertn. (Bing pian). Chemical studies have revealed that the 36 components of SJP include 6 phenolic acids (such as ferulic acid and vanillic acid), 28 phthalides (such as ligustilide and senkyunolide A), ligustrazine and *Borneolum Syntheticum* were identified through the ultra-performance liquid chromatography/quadrupole time-of-flight mass spectrometry (UPLC/Q-TOF MS) [9, 10]. We determined 8 components of SJP which reflected in Additional file 1: Table S1. SJP is broadly utilized in emergency and daily TCM-based therapy of CVD, including angina pectoris and myocardial infarction [11, 12]. Pharmacological studies have shown that SJP has multiple effects, including dilating coronary arteries, dilating vascular smooth muscle, reducing myocardial ischemia, protecting cardiomyocytes, inhibiting the formation of atherosclerotic plaques, reducing blood viscosity and significantly relieving spasm and analgesia [13]. Our previous clinical study confirmed that SJP alleviates major adverse cardiovascular events and improves heart function in acute coronary syndrome (ACS) patients with early revascularization [14], suggesting SJP may mitigate MIRI [15]. Despite its extensive clinical use in China, only few studies have reported the detailed effects of SJP on MIRI [15], and the underlying mechanisms of these effects are undefined.

Here, we used an integrated approach in a rat model of MIRI, together with a hypoxia/reoxygenation (H/R) model in H9c2 cells, to examine the important role of SJP in alleviating MIRI. Furthermore, unveiling the molecular mechanism of SJP in reversing MIRI through m6A methylation modification-mediated autophagy may be a promising target for MIRI treatment.

## Material and methods

### Preparation of SJP

SJP was obtained from the Sixth Chinese Drugs Factory of Tianjin Zhongxin Pharmaceutical (China, Batch No. 617139). The SJP quality control adhered to the specifications and test procedures as described in the Pharmacopoeia of the People’s Republic of China (Pharmacopoeia of the People’s Republic of China, Suxiao Jiuxin Pills, 2015), and the quality control report were provided in Additional file 1: Fig. S1–S5.

### Animal model establishment of MIRI

For MIRI surgeries, Wistar wild-type rats (Shanghai Slack Laboratory Animal, China; 180–200 g) were utilized. Male rats underwent anesthesia with 1% phenobarbital sodium and were positioned supine on a heating pad (37 ℃). Following left thoracotomy (between the 4th and 5th ribs), left anterior descending (LAD) coronary artery ligation was carried out with a 7–0 nylon suture. Regional ischemia was reflected by the discoloration of the myocardium distal to the occlusion and elevated ST segment detected by electrocardiography. Ischemia induction used a 30-min coronary occlusion and a 2-h reperfusion. All animal protocols had approval from the Shanghai University of Traditional Chinese Medicine Medical Center Institutional Animal Care and Use Committee (PZSHUTCM210423002, 2021.04.23).

Rats were divided into 5 groups of 6 animals, including the sham, I/R, SJP low-dose (SJP-L, 32 mg·kg^−1^ d^−1^), SJP medium-dose (SJP-M, 64 mg·kg^−1^ d^−1^), and SJP high-dose (SJP-H, 128 mg·kg^−1^ d^−1^) groups. Sham animals underwent intragastric saline administration and the above surgical procedure except for LAD occlusion; the I/R group had intragastric saline administration and I/R surgery. According to previous studies, clinical dosing, and the Meeh-Rubner equation of dose conversion between humans and rats, the SJP dose groups received SJP intragastrical each day for 14 days before I/R surgery.

### Tissue staining and infarct size measurement

After the 2-h reperfusion, heart perfusion with 1 × phosphate-buffered saline (PBS, pH 7.4) was carried out via the aortic cannula. Then, the LAD ligature was tied again. The heart was weighed and sliced horizontally into five slices. After weighing them individually, these slices underwent incubation with 1% 2,3,5-triphenyltetrazolium chloride (TTC; Sigma, T8877) in 1 × PBS for 8 to 15 min at 37 ℃ and fixation with 10% neutral buffered formalin for 24–48 h. Images were captured under a microscope. TTC—positive (stained red; risk region [RR]) and negative (no red staining; infarct region [IR]) areas were assessed digitally. Infarct size (%) was calculated by the following formula: infarct sizes (%) = IR / left ventricular (LV) area × 100 [16].

### Terminal deoxynucleotidyl transferase-mediated dUTP-biotin nick end labeling (TUNEL) staining

Apoptosis was assessed with a TUNEL staining kit (Service bio, GDP1042), as instructed by the manufacturer. In brief, myocardial tissue sections underwent a 20-min fixation with 4% paraformaldehyde and incubation with 20 µg/mL proteinase K for 40 min at ambient. Upon treatment with 0.1% Triton X-100 for 30 min, incubation was carried out with the TUNEL reaction mixture for 1 h, shielded from light. Finally, a BX51 inverted fluorescence microscope (Olympus) was used to count apoptotic cells in 4 random high-power fields per specimen.

### Echocardiography

Echocardiograms were obtained on a Vevo 2100 system (MS400C probe) in the conscious state after gently restraining the rats followed by 24 h reperfusion, by sonographers blinded to grouping. Left ventricular end-diastolic (LVEDD) and end-systolic (LVESD) dimensions were derived from M-mode images. FS (in %) was derived as (LVEDD − LVESD)/LVEDD. Measurements were performed at the level of papillary muscles per standard protocols.

### Cell lines and culture conditions

H9c2 cardiomyocytes underwent culture in Dulbecco's modified eagle media (DMEM; Gibco, 11965092). Then, the cells were seeded in a 6-well plate at 2 × 10^6^/well and incubated in DMEM containing 10% fetal bovine serum (FBS; Gibco, 10099141) and penicillin/streptomycin (100 mg/mL, Thermo Fisher Scientific, 15140163). Following a 24-h culture, complete medium was replaced with freshly prepared DMEM supplemented with cardiomyocyte growth supplements at 37℃ in presence of 5% CO2. Next, H9c2 cells were cultured with reoxygenation (95% air and 5% CO_2_) for 4 h after hypoxia (1% O_2_, 94% N_2_ and 5% CO_2_) for 2 h (Additional file 1: Fig. S6). The medium was replaced daily.

### Drug preparation and cell treatment

According to previous experiments, the two major components of SJP are tetramethylpyrazine (TMP) and borneol (BOR) [11]. To assess SJP’s cardioprotective properties after H/R injury in H9c2 cells, 7 groups (*n* = 3 per group) were examined, including the blank, H/R (H/R + 0.1% ethanol), SJP (H/R + 50 mg/mL SJP), CHIR99021 (H/R + 50 mg/mL SJP + 1 µM CHIR99021), rapamycin (H/R + 50 mg/mL SJP + 0.1 µM rapamycin), TMP (H/R + 100 mg/mL TMP), and BOR (H/R + 25 mg/mL BOR) groups. The dosages of all drugs for cell culture are based on cell counting kit 8 (CCK8) assay (Additional file 1: Fig. S7). SJP, TMP and BOR were diluted in 0.1% ethanol to 50 g/mL, 100 g/mL and 25 g/mL, respectively, as 1000 × stock solutions for the cell culture experiments. CHIR99021 (Selleck, S1263) and rapamycin (Selleck, S1039) were added to the incubation medium to block the expression of GSK3β and mTOR, respectively. Pretreatment with all drugs for 1 h was performed before H/R modeling in H9c2 cells.

To define the function of m6A modification in SJP-regulated autophagy in H/R-induced H9c2 cells, 5 groups (*n* = 3 per group) were examined, including the blank, H/R (H/R + 0.1% ethanol), SJP (H/R + 50 mg/mL SJP), TMP (H/R + 100 mg/mL TMP), and BOR (H/R + 25 mg/mL BOR) groups. H9c2 cells in each 6 well plate were treated with 0.1% ethanol, 50 mg/mL SJP, 100 mg/mL TMP or 100 mg/mL BOR for 1 h before H/R modeling.

To determine whether ALKBH5 and GSK3β/mTOR signaling played crucial roles in SJP-dependent autophagy, 12 groups (*n* = 3 per group) were examined, including the shRNA-Ctrl + H/R, shRNA-Ctrl + SJP (shRNA-Ctrl + H/R + 50 mg/mL SJP), shRNA-Ctrl + TMP (shRNA-Ctrl + H/R + 100 mg/mL TMP), shRNA-Ctrl + BOR (shRNA-Ctrl + H/R + 25 mg/mL BOR), shRNA-ALKBH5 + H/R, shRNA-ALKBH5 + SJP, shRNA-ALKBH5 + TMP, shRNA-ALKBH5 + BOR, shRNA-GSK3β + H/R, shRNA-GSK3β + SJP, shRNA-GSK3β + TMP, and shRNA-GSK3β + BOR groups. The drugs were added in the incubation medium of transfected cells for 1 h before H/R modeling in H9c2 cells.

To further confirm whether GSK3β is crucial in ALKBH5-dependent autophagy after SJP treatment, 8 groups (*n* = 3 per group) were examined, including the shRNA-ALKBH5 + H/R, shRNA-ALKBH5 + SJP, shRNA-ALKBH5 + TMP, shRNA-ALKBH5 + BOR, shRNA-ALKBH5 + GSK3β-inhibitor (1 µM CHIR99021) + H/R group, shRNA-ALKBH5 + GSK3β-inhibitor + SJP, shRNA-ALKBH5 + GSK3β-inhibitor + TMP, and shRNA-ALKBH5 + GSK3β-inhibitor + BOR groups. Both CHIR99021 and the drugs were added to the transfected cells for 1 h before H/R modeling in H9c2 cells.

### Annexin V/propidium iodide (PI) assay

Apoptotic cells were quantitated with an Annexin V-FITC/PI apoptosis detection kit (Multi Sciences Biotech, AP101-100), as directed by the manufacturer. In brief, after staining of H9c2 cells with Annexin V and PI for 30 min at ambient, a FACScan flow cytometer (BD FACSAria, USA) was utilized for analysis within 1 h.

### Adenovirus-mediated gene transfer

H9c2 myocytes underwent infection 72 h post-seeding with adenovirus harboring RFP-GFP-tagged LC3 (designed by the Hill Lab at UT Southwestern), at a multiplicity of infection (MOI) of 30 PFU/cell. Subsequently, the cells underwent culture under reoxygenation for 4 h after hypoxia for 2 h.

### Transmission electron microscopy (TEM)

Harvested cells underwent PBS washing and were fixed with 2.5% glutaraldehyde at 4 °C overnight. This was followed by three washes with 0.1 M PBS and incubation with 1% citric acid (Pelco, USA) at 4 °C for 3 h. After another PBS wash, dehydration was performed with an ethanol gradient; next, propylene oxide was added. The Spurr resin (Spi-Chem; Structure Probe, USA) was utilized for embedding, with polymerization occurring at 70 °C. Next, 70-nm sections were obtained on an ultrathin slicer (Leica, EM UC6), and underwent staining with uranyl acetate and lead citrate (Spi-Chem, Structure Probe). A transmission electron microscope (JEOL Ltd., JEM1230) was utilized for analysis.

### ShRNA knockdown

H9c2 cardiomyocytes were isolated and seeded at 1.2 × 10^6^/well in 6-well plates. At 24 h post-plating, cardiomyocytes underwent incubation with short hairpin RNA (shRNA) negative control (Neg, SIC001), shRNAs targeting ALKBH5 (VL1931-PDS125_pL-U6-SHRNA-ALKBH5-599, F-CACCGCTGCATCGTATCTCACGTAGCGAACTACGTGAGATACGATGCAGC and R- AAAAGCTGCATCGTATCTCACGTAGTTCGCTACGTGAGATACGATGCAGC), and shRNAs targeting GSK3β (VL1938-PDS125_pL-U6-SHRNA-GSK3b-596, F-CACCGCGACTTTGGAAGTGCAAAGCCGAAGCTTTGCACTTCCAAAGTCGC and R- AAAAGCGACTTTGGAAGTGCAAAGCTTCGGCTTTGCACTTCCAAAGTCGC) as directed by the manufacturer.

### Quantification of m6A modification

Total RNA isolation utilized TRIzol (Invitrogen, 15596018) as instructed by the manufacturer, followed by deoxyribonuclease I (Sigma-Aldrich, 04716728001) treatment. A NanoDrop was employed to assess RNA quality. The altered m6A amounts in mRNA were assessed with a colorimetric EpiQuik m6A RNA Methylation Quantification Kit (Epigentek, P-9005-48) as proposed by the manufacturer. Poly-A-purified RNA (200 ng) was utilized to analyze every specimen [7].

### Quantitative reverse transcription-PCR (qRT-PCR)

Total RNA underwent reverse transcription with the Script One-Step RT-PCR kit (Takara, Japan). Then, qRT-PCR was performed with SYBR Premix EX Taq kit (TaKaRa, RR420A) on an ABI 7500 instrument (Applied Biosystems). The 2^−ΔΔCt^ method was employed for analyzing triplicate data normalized to GAPDH expression. The primers used were: ALKBH5-F 5′-GACCTGCGTGAGAAGCTCAA-3′and ALKBH5-R 5′-TGGTACTTCCGTTTGGTGGTC-3′.

### Western blotting analysis

Primary antibodies targeting LC3B (1:1000; Abcam, ab221794, 14, 16 kDa), Atg5 (1:1000; Abcam, ab108327, 55 kDa), Atg7 (1:1000; Abcam, ab133528, 77 kDa), p62 (1:1000; Abcam, ab109012, 62 kDa), ALKBH5 (1:1000; Abcam, ab195377, 44 kDa), p-GSK3β (Ser9, 1:1000; Abcam, ab107166, 47 kDa), GSK3β (1:1000; Abcam, ab280376, 46 kDa), p-mTOR (S2448, 1:1000; Abcam, ab109268, 289 kDa), mTOR (1:1000; Abcam, ab134903, 289 kDa), and Beclin-1 (1:1000; Abcam, ab207612, 52 kDa) were used. Horseradish peroxidase (HRP)-linked anti-mouse IgG and anti-rabbit IgG (1:5000; Protein Tech Group, SA00001-2) were used, respectively. Data were normalized to β-actin expression. Immunoreactive bands were quantified by densitometry and computer-assisted image analysis.

### Statistical analysis

Data are mean ± standard error of the mean (SEM). One-way analysis of variance (ANOVA) was carried out for comparing multiple groups, with post hoc Bonferroni test, using SPSS 21.0 (IBM, USA). *P* < 0.05 was deemed to be statistically significant. GraphPad Prism 7.0 (GraphPad Software, USA) was utilized for graphing.

## Results

### SJP inhibited MIRI and cardiomyocyte apoptosis in rats

**Fig. 1:**
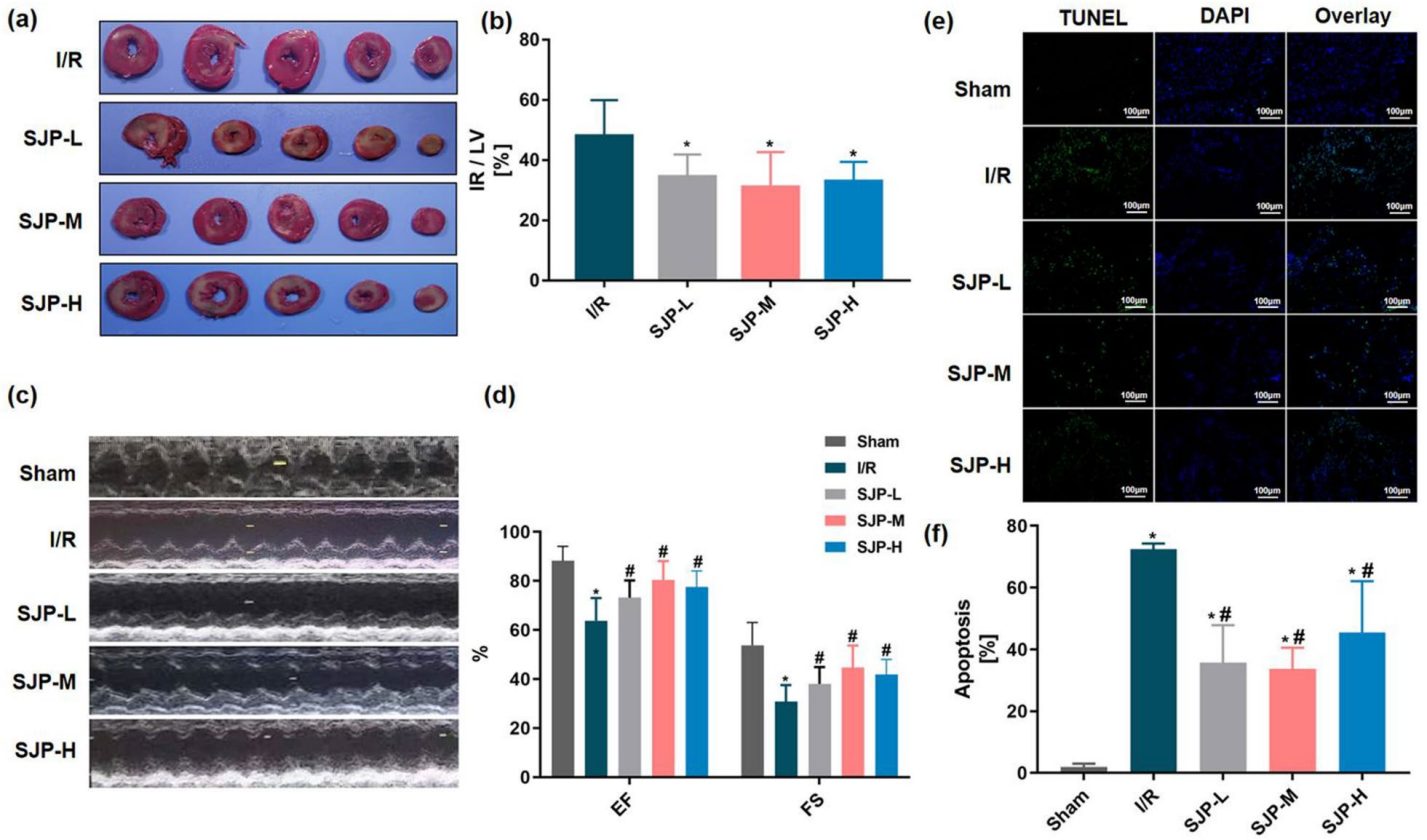
SJP inhibits MIRI and cardiomyocyte apoptosis in rats (*n* = 6). **a** and **b** SJP reduced the IR zone in the TTC assay. Data are mean ± SEM. *P < 0.05 versus I/R group. **c** and **d** SJP improved heart function in rats as examined by echocardiography. *P < 0.05 versus Sham group; #*P* < 0.05 versus I/R group. **e** and **f** SJP decreased apoptosis in myocardial cells in rats as assessed by TUNEL assay (× 200). *P < 0.05 versus Sham group; #*P* < 0.05 versus I/R group

As shown in Fig. 1a, b, I/R was differentiated from the RR zone. The ratio of IR/LV was significantly lower in the SJP groups than in the I/R group (*P* < 0.05), but no significant differences were observed in SJP groups while the SJP medium-dose group reduced the I/R zone markedly. The therapeutic effect of drugs on MIRI is also reflected in the improvement of cardiac function, and hence, the effect of SJP was evaluated through an electrocardiograph. The SJP groups significantly increase the levels of LVEF and LVFS in MIRI rats compared to the I/R group (*P* < 0.05, Fig. 1c, d). No statistical difference was detected among the dose groups, while the medium-dose group had a trend of better efficacy. Sustained myocardial ischemia induces a large number of myocardial cell apoptosis, mainly in the peripheral area of myocardial infarction [17]. TUNEL staining was used to detect the apoptosis level of myocardial cells under the intervention of SJP. Compared to the sham group, the apoptosis of I/R and SJP groups was increased significantly (*P* < 0.05, Fig. 1e, f), and the apoptosis ratios were reduced after different-dose SJP administration. Furthermore, the medium-dose group had a significant effect (*P* < 0.05). Taken together, these results indicated that SJP inhibits MIRI in rats.

### SJP inhibited autophagy through GSK3β/mTOR pathway in MIRI rats

**Fig. 2:**
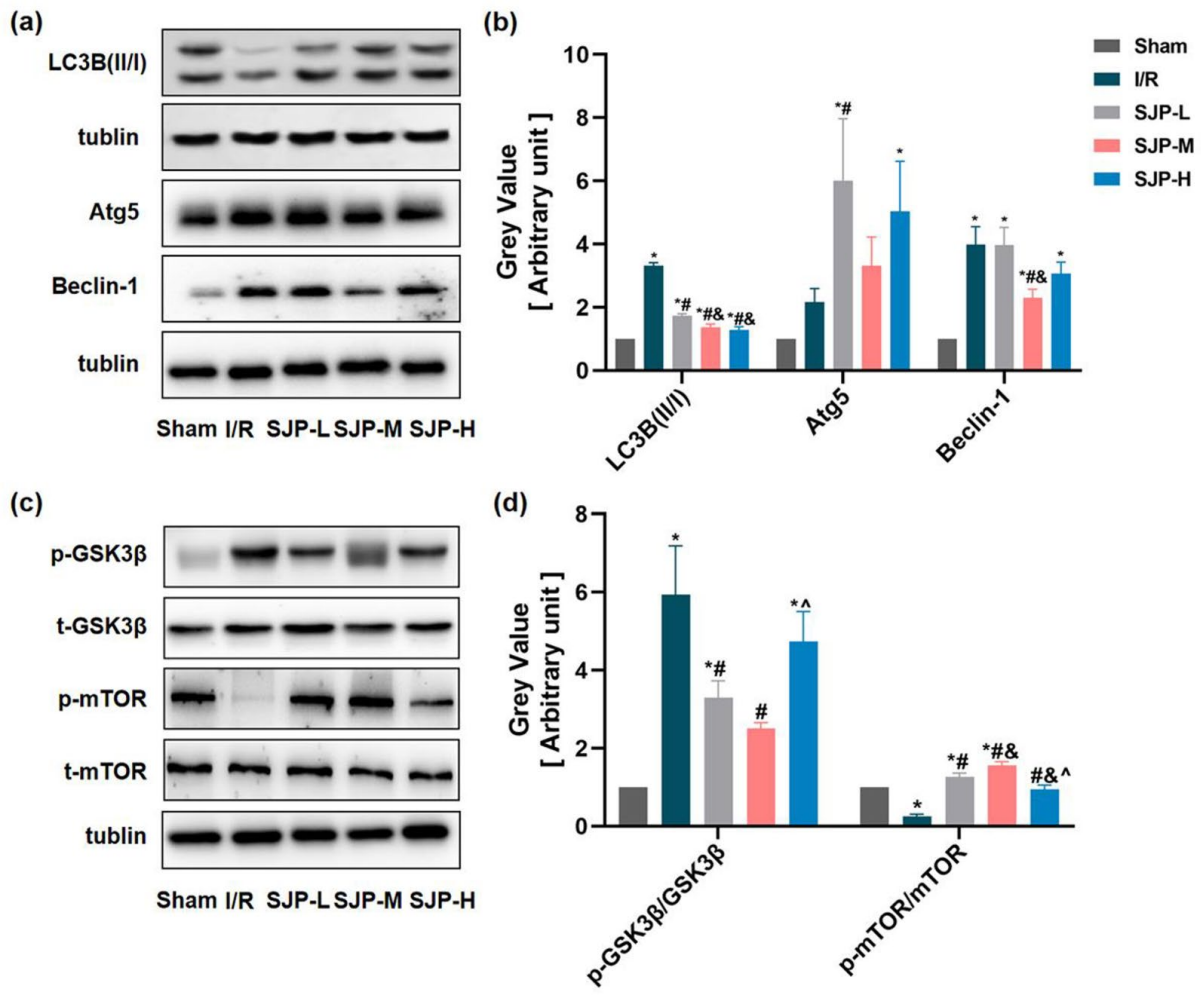
SJP inhibits autophagy through the GSK3β/mTOR pathway in MIRI rats(*n* = 6). **a **and **b**SJP inhibited myocardial autophagy in rats as assessed by western blot. **c** and **d** SJP regulated p-GSK3β/GSK3β and p-mTOR/mTOR in rats as determined by western blot. Data are mean ± SEM. *P < 0.05 versus Sham group; #P < 0.05 versus I/R group; &*P* < 0.05 versus SJP-L group; ^*P* < 0.05 versus SJP-M group

Under the condition of cardiac I/R injury, the process of autophagy is activated in response to the energy crisis and oxidative stress. Then, we assessed the effect of SJP on the autophagy marker LC3B and autophagy-related molecules, including Atg5 and Beclin-1 (Atg12) in MIRI rats by western blot. As shown in Fig. 2a, b, increased LC3B-II:LC3B-I ratio and Beclin-1 levels were observed in our I/R group (*P* < 0.05). Compared to the I/R group, the SJP groups significantly decreased the levels of LC3B II/I and Beclin-1 (*P* < 0.05). However, Atg5 did not show the same trend. Together, these results suggested that SJP inhibits autophagy in MIRI rats.

GSK-3 is a serine/threonine kinase involved in many cellular functions in the heart, including gene expression, hypertrophy, and apoptosis [18, 19]. Also, it is a target of drug therapy for I/R injury because phosphorylation/inhibition of mitochondrial GSK-3β during I/R might be a common mechanism mediating myocardial protection by many agents/interventions [20–22]. Moreover, GSK3β inhibition increases cardiac tolerance to MIRI due to autophagy inhibition [20]. In previous studies, we revealed that SJP has a specific effect on GSK3β in hypoxic cardiomyocytes [23]. Since GSK3β overexpression induces autophagy by inhibiting the activity of mTOR [24], we explored the effect of SJP on the expression of GSK3β and mTOR in MIRI rats by western blot. As shown in Fig. 2c, d, the level of p-GSK3β/GSK3β was significantly upregulated while p-mTOR/mTOR was downregulated when rats were exposed to the I/R condition and SJP groups altered the expression of these two proteins compared to the I/R group. In the current study, the effect of SJP on GSK3β expression in MIRI rats was consistent with the results of Tan et al. [15]. Furthermore, our results revealed that SJP inhibits autophagy through GSK3β/mTOR pathway in MIRI rats.

### SJP inhibited autophagy through GSK3β/mTOR pathway with gene inhibitor in H/R-induced H9c2 cardiomyocytes

**Fig. 3:**
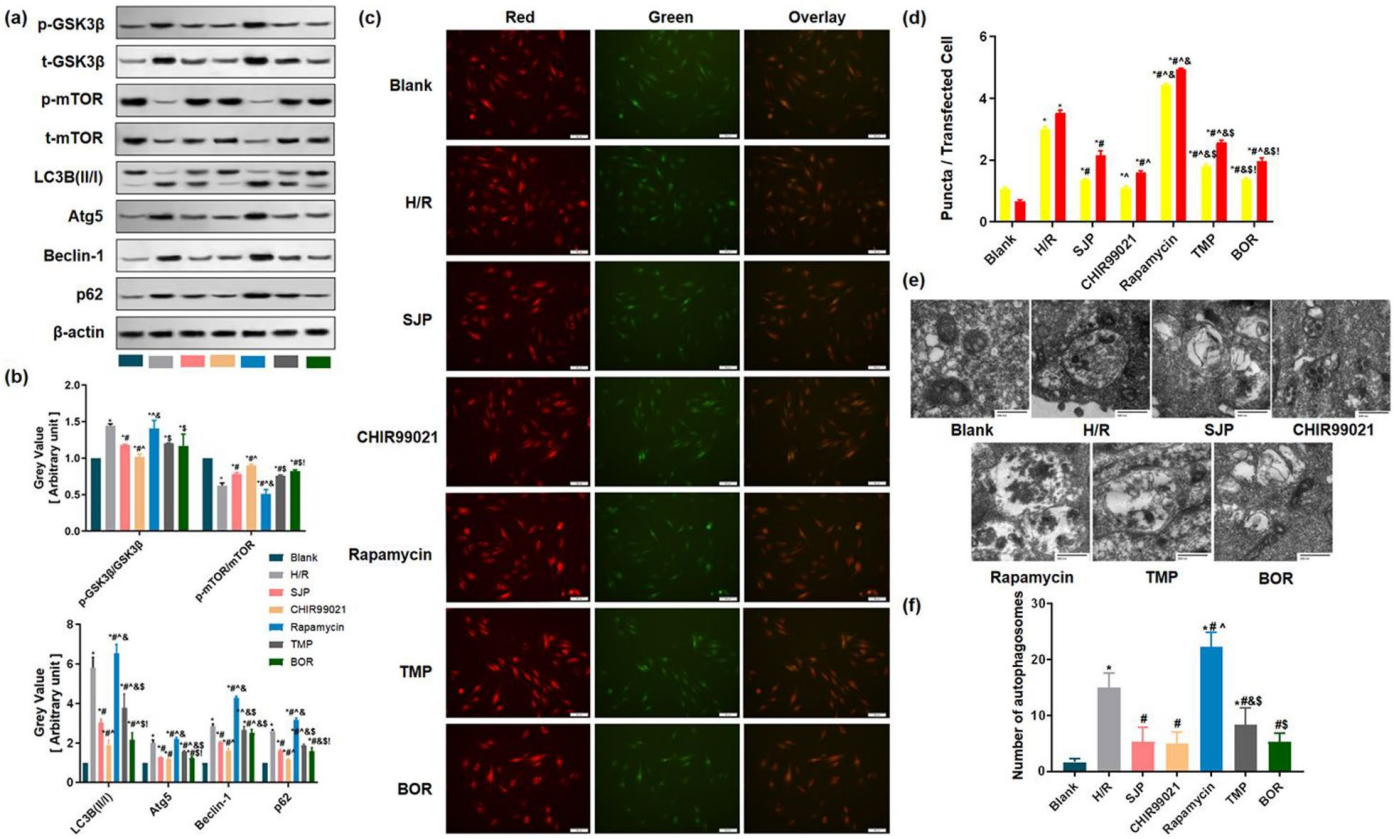
SJP inhibits autophagy through the GSK3β/mTOR pathway with gene inhibition assays in H/R-treated H9c2 cardiomyocytes (*n* = 3). **a** and **b** SJP inhibited myocardial autophagy in rats as assessed by western blot. **c** and **d** H9c2 cells underwent transfection with adenoviruses harboring tandem fluorescent mRFP-GFP-LC3 (Ad-LC3-H9c2s) for 24 h and various treatments. Representative images of immunofluorescent H9c2 cells expressing mRFP-GFP-LC3 are depicted (bar = 50 μm). GFP and mRFP signals are green and red, respectively. Semiquantitative assessment of autophagosomes (AP, yellow dots in merged images) and autolysosomes (AL, red dots in merged images). **e** and** f** Numbers of autophagosomes determined by transmission electron microscopy (TEM, × 40,000). Data are mean ± SEM. ^*^P < 0.05 versus Blank group; ^**#**^*P* < 0.05 versus H/R group; ^^^*P* < 0.05 versus SJP group; ^&^*P* < 0.05 versus CHIR99021 group; ^$^*P* < 0.05 versus Rapamycin group; ^!^*P* < 0.05 versus TMP group

Firstly, to determine whether the effect of SJP and its active components (TMP and BOR) inhibiting autophagy were involved in H/R-induced cardiomyocytes injury, H9c2 cells were exposed to H/R. Consistent with the results in MIRI rats, the levels of LC3B II/I, Atg5, and Beclin-1 were elevated after H/R but were reversed under the intervention of SJP (Fig. 3a, b). Secondly, since SQSTM1 (sequestosome 1, p62) is negatively correlated with autophagic flux [25], we assessed the effect of SJP on p62 levels. As shown in Fig. 3a, p62 is enhanced in the H/R group, and SJP inhibits the expression of p62 in H/R-induced H9c2 cell injury (*P* < 0.05). Thirdly, tandem fluorescent mRFP-GFP-LC3 transfection was performed in H9c2 (AdLC3-H9c2) [26]. Normal Ad-LC3-H9c2 had basal autophagy with few autolysosomes (red only puncta) and few autophagosomes (yellow puncta) (Fig. 3c). Autolysosomes and autophagosomes were elevated in the H/R group, and the trend was suppressed under SJP control. Finally, in terms of morphology, when autophagy occurs, a large number of free membranous structures appear in the cytoplasm, which continuously expands and wrap around the substances to be degraded to form autophagosomes [27]. The autophagosome structure observed under TEM is the gold standard for proving autophagy [26]. SJP reduced the number of autophagosomes in H/R-induced H9c2 cells. Taken together, these results indicated that SJP inhibits autophagy in MIRI rats and H/R-induced H9c2 cells.

Consistent with the results of I/R in rats (Fig. 2a), the trend of p-GSK3β/GSK3β and p-mTOR/mTOR was similar in the cardiomyocytes after H/R (Fig. 3a, b).

To further explore the role of GSK3β and mTOR in H/R-induced H9c2 cells with SJP administration, we used CHIR99021 and rapamycin to reduce the expression of GSK3β and mTOR, respectively, in H9c2 cells. In the current experiments, the addition of CHIR99021 further inhibited p-mTOR/mTOR, LC3B II/I, Atg5, Beclin-1, and p62 levels, as well as autolysosomes and autophagosomes compared to the SJP group (Fig. 3). Moreover, autophagy was reversed after rapamycin compared to the SJP group. Collectively, these results revealed that SJP inhibits autophagy through GSK3β/mTOR pathway in MIRI rats and H/R-induced H9c2 cells.

### SJP stimulated ALKBH5 expression in H/R cardiomyocytes, which accounted for the aberrant m6A modification

**Fig. 4:**
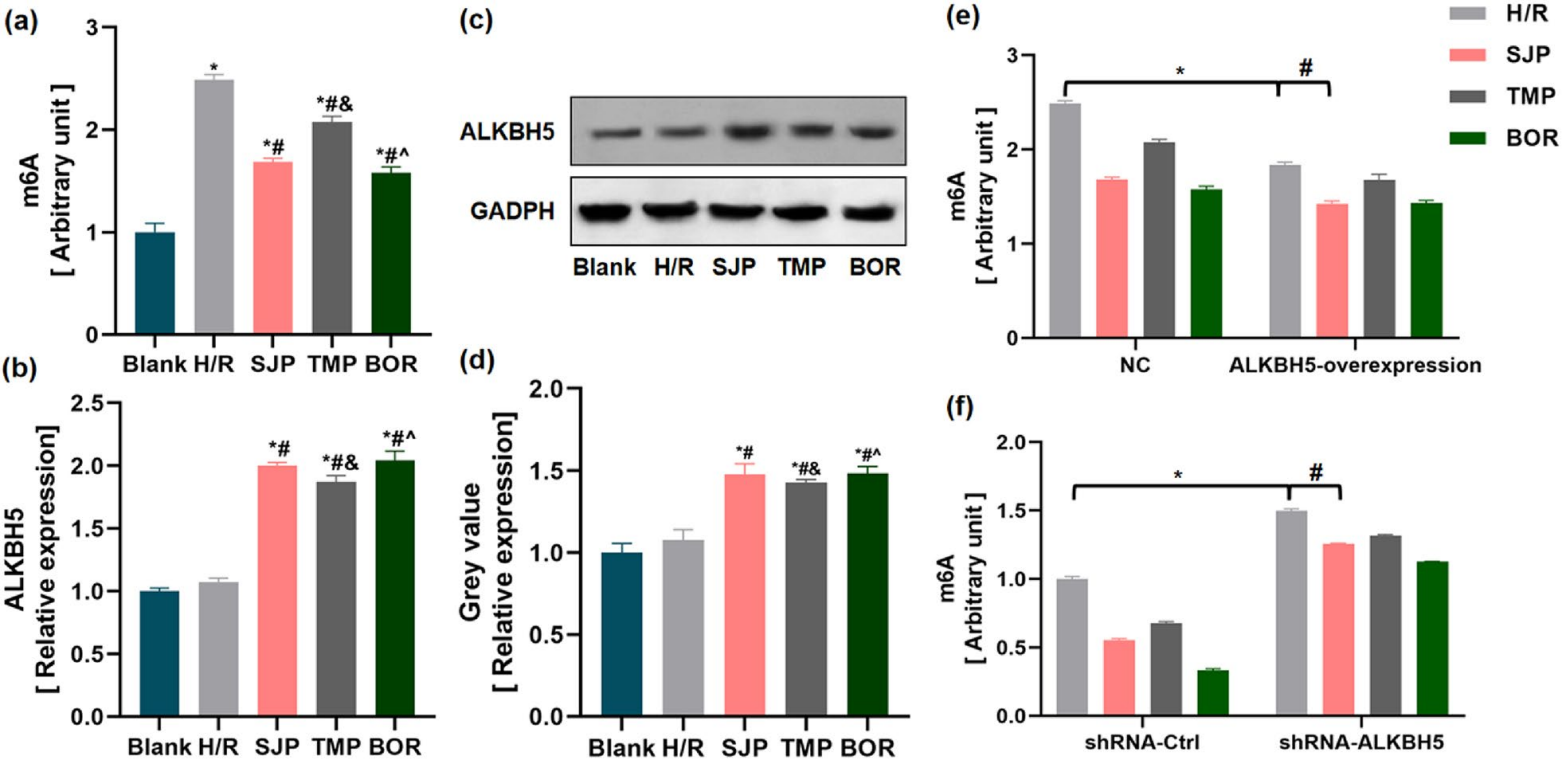
SJP induces ALKBH5 expression in H/R cardiomyocytes, resulting in aberrant m6A modification (*n* = 3). **a** SJP inhibited m6A modification in H/R-treated H9c2 cells. **b** ALKBH5 mRNA amounts in H/R-treated H9c2 cells by PCR. **c** and **d** ALKBH5 protein amounts in H/R-treated H9c2 cells assessed by immunoblot. Data are mean ± SEM. ^*^P < 0.05 versus Blank group; ^**#**^*P* < 0.05 versus H/R group; ^&^*P* < 0.05 versus SJP group; ^^^*P* < 0.05 versus TMP group. **e** m6A modification levels in the ALKBH5 overexpression assay. ^*^P < 0.05 versus NC + H/R group; ^#^*P* < 0.05 versus ALKBH5-overexpression + H/R group. **f** m6A modification levels after transfection with ALKBH5 shRNA. ^*^P < 0.05 versus shRNA-Ctrl + H/R group; ^#^*P* < 0.05 versus shRNA-ALKBH5 + H/R group

To define the role of m6A modification in SJP-regulated autophagy in H/R induced H9c2 cells, we performed m6A quantification analysis of mRNAs in H/R-induced H9c2 cells and found that the levels of m6A modification were significantly increased in H9c2 cells following H/R (Fig. 4a). This phenomenon indicated that m6A levels were dynamically regulated when cardiomyocytes were treated by H/R, while SJP, TMP, and BOR reversed the levels of m6A modification; also, SJP and BOR were superior to TMP in reducing the m6A modification.

The m6A modification is primarily catalyzed by an m6A methyltransferase complex containing METTL3, METTL14, WTAP, and an m6A demethyltransferase (FTO, ALKBH5) [28]. Next, we hypothesized that the abnormal m6A modification in cardiomyocytes after H/R was caused by the dysregulation of key m6A methyltransferase and demethytransferase members. The forced expression of ALKBH5 via adenoassociated virus-9 (AAV9) delivery reduces the infarct size, and the cardiac function is restored after myocardial infarction in juvenile (7-days-old) and adult (8-weeks-old) mice [29]. Furthermore, Zhu et al. found that ALKBH5 activated mTOR signaling pathway in epithelial ovarian cancer [30]. Thus, we hypothesized that ALKBH5 plays a significant role in SJP-regulated GSK3β/mTOR signaling pathway in H/R-induced H9c2 cells. To investigate this theory, we measured the gene and protein expression levels of ALKBH5 in H9c2 cells. Interestingly, ALKBH5 was not altered significantly in H/R-induced H9c2 cells but increased in H/R-induced H9c2 cells after SJP, TMP, and BOR administration (Fig. 4b–d). These results implied that ALKBH5 plays a regulatory role in SJP-mediated m6A modification. To test this possibility, ALKBH5 silencing and overexpression experiments were used in H/R-induced H9c2 cells. ALKBH5 overexpression reduced m6A modification, while transfection of ALKBH5 shRNA significantly enhanced m6A modification in the mRNA in the H/R group. Strikingly, SJP, TMP, and BOR addition reduced the level of m6A modification in H/R + ALKBH5-overexpression and H/R + shRNA-ALKBH5 groups (Fig. 4e, f). These data indicated that ALKBH5 exerts a regulatory role in SJP-mediated m6A demethylation.

### SJP inhibited autophagic activity through ALKBH5 and GSK3β/mTOR pathway in gene knockout experiments following H/R

To determine whether ALKBH5 plays a crucial role in SJP-dependent autophagy, we overexpressed ALKBH5 in H/R-induced H9c2 cells after SJP administration. Consistent with upregulation of ALKBH5, *GSK3β* mRNA and autophagy-related genes were dramatically downregulated and *mTOR* mRNA was upregulated in H/R-induced cardiomyocytes with SJP pretreatment (Additional file 1: Fig. S8 and Additional file 1: Table S2). The results suggested that upregulation of ALKBH5 impaired autophagy and promoted apoptosis in H/R-induced cardiomyocytes.

**Fig. 5:**
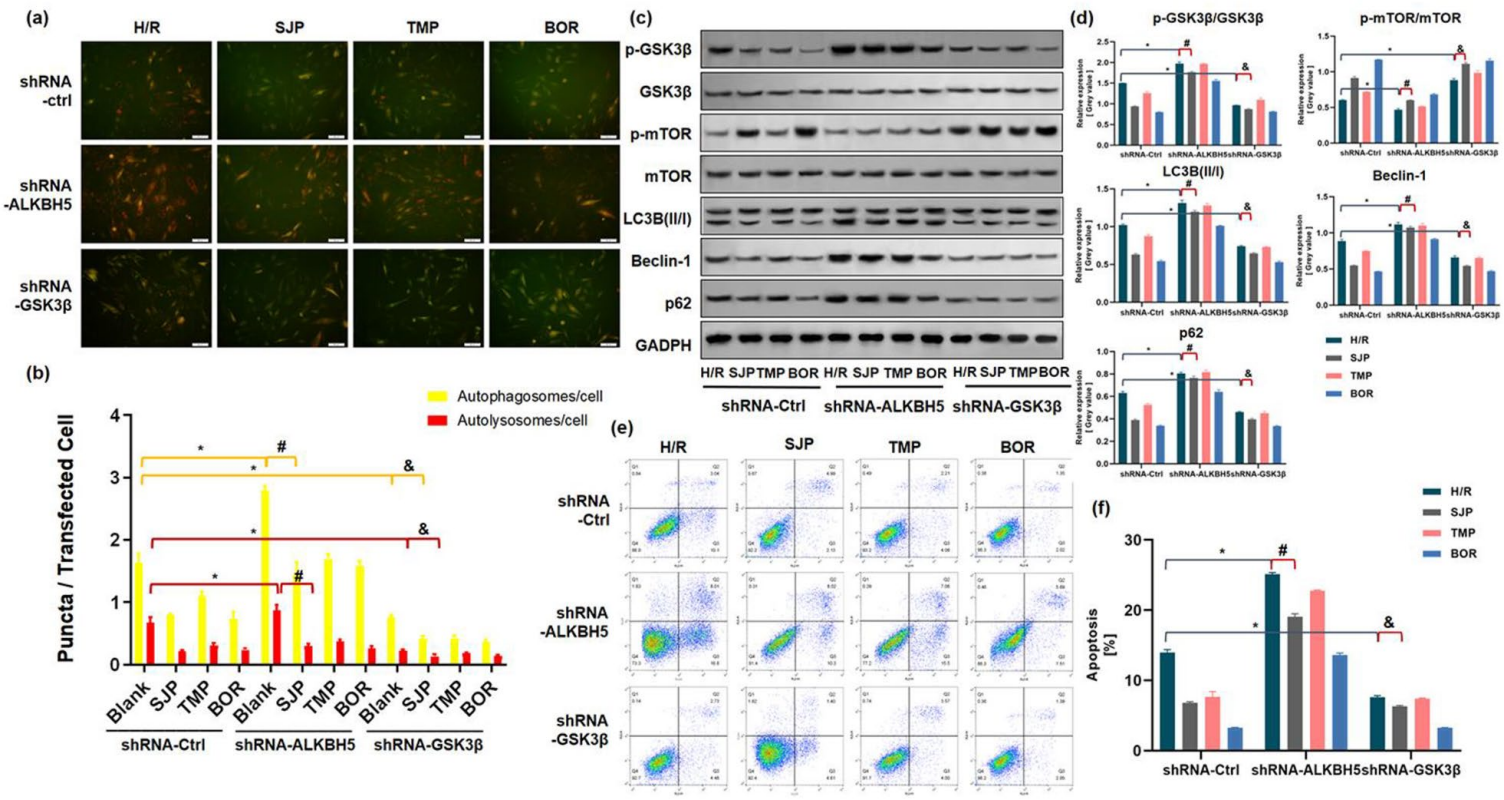
SJP inhibits autophagic activity through the ALKBH5 and GSK3β/mTOR pathway in gene knockout experiments following H/R (*n* = 3). **a** and **b** H9c2 cardiomyocytes underwent transfection with adenovirus carrying tandem fluorescent mRFP-GFP-LC3 (Ad-LC3-H9c2s) for 24 h and various treatments (bar = 50 μm). **c** and **d** The protein amounts of p-GSK3β/GSK3β, p-mTOR/mTOR, LC3B (II/I), Beclin-1, and p62 in H/R-treated H9c2 cells by western blot. **e** and **f** Apoptotic rates of cardiomyocytes in H/R-induced H9c2 cells assessed by the Annexin V/PI assay. Data are mean ± SEM. ^*^P < 0.05 versus shRNA-Ctrl + H/R group; ^#^ *P* < 0.05 versus shRNA-ALKBH5 + H/R group; ^&^*P* < 0.05 versus shRNA-GSK3β + H/R group

Then, we conducted ALKBH5-knockdown and GSK3β-knockdown experiments in H/R-induced H9c2 cells (Additional file 1: Fig S9). The silencing of ALKBH5 enhanced the autophagy and the expression of p-GSK3β/GSK3β and weakened the level of p-mTOR/mTOR in H/R cardiomyocytes. Subsequently, SJP, TMP, and BOR reversed the autophagy and the levels of p-GSK3β/GSK3β and p-mTOR/mTOR, which was contrary to the results of ALKBH5 overexpression experiments. The knockdown of GSK3β weakened the autophagic activity in H/R cardiomyocytes, which was reversed with the administration of SJP, TMP, and BOR (Fig. 5a–d). This finding was consistent with the results of GSK3β inhibition in H/R-induced H9c2 cells (Fig. 5a–f). Interestingly, in our gene knockout experiments, BOR inhibited the autophagic activity maximally compared to other agents.

Owing to the complex correlation between autophagy and apoptosis regulation of cell death [31], we examined the effect of ALKBH5-knockdown and GSK3β-knockdown on cardiomyocyte apoptosis. The silencing of ALKBH5 enhanced the apoptosis rate, and SJP, TMP, and BOR reduced apoptosis. The knockdown of GSK3β weakened the apoptosis activity in H/R cardiomyocytes, and the administration of SJP, TMP, and BOR reversed the trend (Fig. 5e, f). Together, these results indicated that SJP inhibits the autophagic activity through ALKBH5 and GSK3β/mTOR pathway in H/R-induced cardiomyocytes.

### SJP inhibited excessive autophagy through ALKBH5/GSK3β pathway in H/R-induced H9c2 cells

**Fig. 6:**
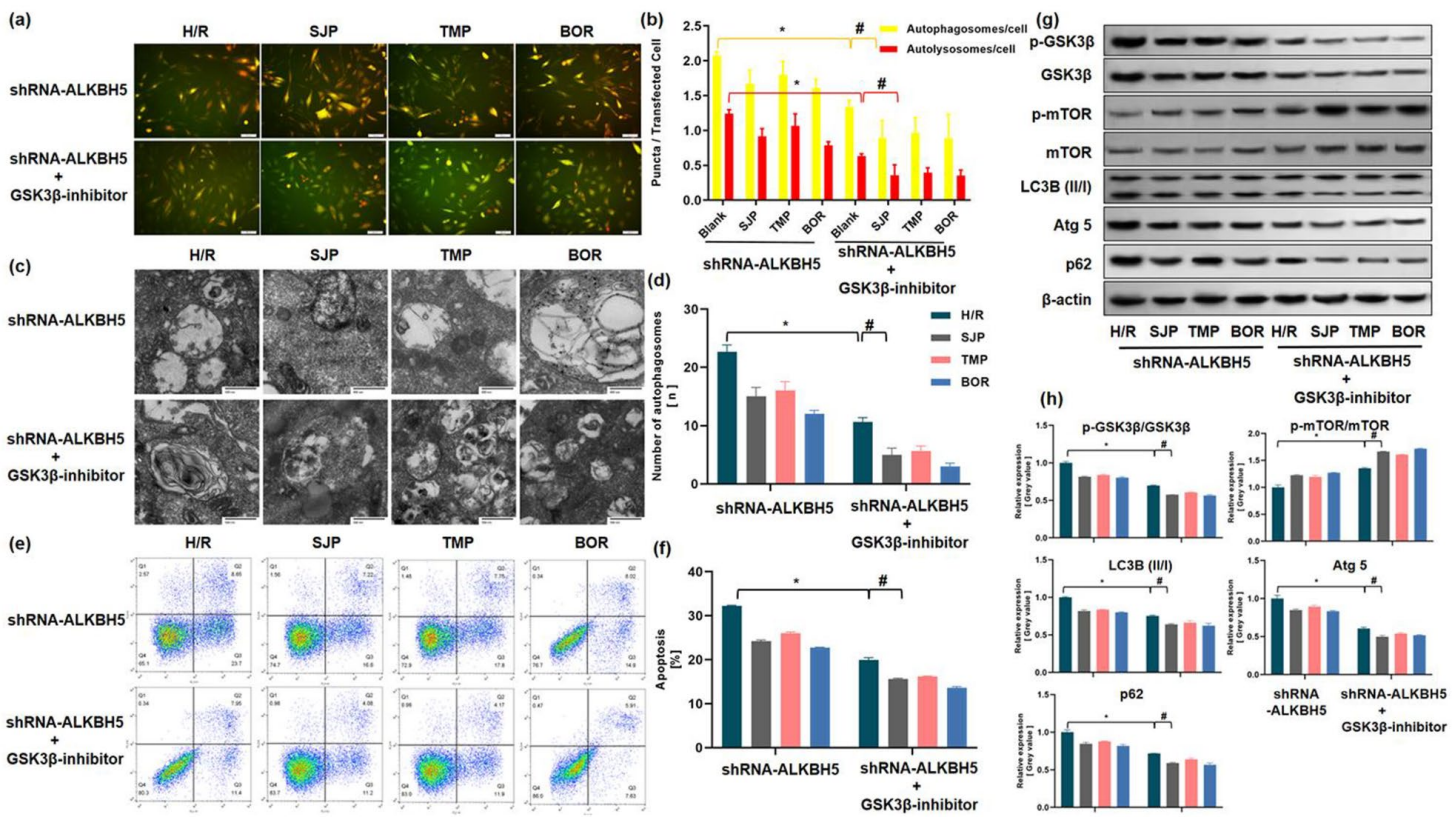
SJP inhibits excessive autophagy through the ALKBH5/GSK3β pathway in H/R-induced H9c2 cells (*n* = 3). **a** and **b** H9c2 cardiomyocytes were transfected with adenoviruses harboring tandem fluorescent mRFP-GFP-LC3 (Ad-LC3-H9c2s) for 24 h and subjected to various treatments (bar = 50 μm). **c** and **d** Numbers of autophagosomes determined by TEM (× 40,000). **e** and **f** Apoptotic rates of H/R-treated H9c2 cells assessed by the Annexin V/PI assay. **g** and **h** Protein levels of p-GSK3β/GSK3β, p-mTOR/mTOR, LC3B (II/I), Atg5, and p62 in H/R-treated H9c2 cells evaluated by western blot. Data are mean ± SEM. ^*^P < 0.05 versus shRNA-ALKBH5 + H/R group; ^#^*P* < 0.05 versus shRNA-ALKBH5 + GSK3β-inhibitor + H/R group

ALKBH5 activated mTOR signaling pathway in epithelial ovarian cancer [30]. Based on our results of ALKBH5 and GSK3β knockdown experiments, we hypothesized that SJP protects MIRI by inhibiting autophagy of cardiomyocytes through ALKBH5 regulation of GSK3β/mTOR pathway. To further confirm whether GSK3β plays a crucial role in ALKBH5-dependent autophagy after SJP treatment, we conducted an experiment to assess whether GSK3β-inhibitor reverses the shRNA-ALKBH5-mediated autophagy in H/R-induced H9c2 cells after SJP treatment. The current results showed that the inhibition of GSK3β in shRNA-ALKBH5 cells reversed the subcellular redistribution of GFP-LC3 (Fig. 6a, b), retarded the number of autophagosomes (Fig. 6c, d), weakened apoptosis activity (Fig. 6e, f), inhibited autophagic activity with LC3B (II/I), Atg5, and p62, lowered the expression of p-GSK3β/GSK3β level, and elevated p-mTOR/mTOR mediated by H/R-induced H9c2 cells (Fig. 6g, h). With the administration of SJP, TMP, and BOR the above trend was retarded; among these, BOR showed a maximal effect. In summary, these results suggested that SJP inhibits autophagic activity to protect H/R-induced H9c2 cells through ALKBH5/GSK3β/mTOR pathway.

## Discussion

The present study revealed that SJP alleviates MIRI by inhibiting excessive autophagy. We first demonstrated that SJP alleviated MIRI in rats, likely by suppressing excessive autophagy in the myocardium. SJP and its active constituents also alleviated excessive autophagy in H/R-induced H9c2 cells. Several signaling pathways are involved in autophagy regulation, among which mTOR signaling is the classic pathway [32]. Chen et al. speculated that GSK3β alleviates MIRI by suppressing excessive autophagy in cardiomyocytes [20]. According to our preliminary results, SJP exerted a regulatory effect on GSK3β. It is known GSK3β overexpression induces autophagy by inhibiting mTOR activity [24]. Herein, SJP upregulated mTOR in H/R-induced H9c2 cells and MIRI rats but suppressed autophagy in H/R-treated cardiomyocytes after pharmacological GSK3β inhibition or GSK3β gene knockout. Furthermore, treatment with an mTOR inhibitor promoted the regulatory effect of SJP on autophagy in H/R-induced cardiomyocytes, suggesting SJP inhibits autophagy in cardiomyocytes through the GSK3β/mTOR signaling pathway. It was recently demonstrated m6A methylation is tightly associated with autophagy in MIRI [7]. In this study, m6A was detected in H/R-treated H9c2 cells, and SJP significantly inhibited this modification. Then, we hypothesized that aberrant m6A modification in cardiomyocytes post-H/R results from a deregulation of the major m6A demethyltransferase ALKBH5. ALKBH5, or m6A eraser, is a m6A demethylase that has attracted substantial attention among biologists and pharmacologists [33]. Interestingly, Zhu et al. found that ALKBH5 activates mTOR signaling in epithelial ovarian cancer [30]. Thus, we hypothesized that ALKBH5 plays a significant role in SJP-regulated GSK3β/mTOR pathway in H/R-treated H9c2 cells. To investigate this possibility, ALKBH5 amounts were determined in H9c2 cells. As shown above, ALKBH5 did not change significantly in H/R-treated H9c2 cells in comparison to blank control cells. Interestingly, ALKBH5 expression was increased in H/R-treated H9c2 cells administered SJP, TMP, and BOR, respectively. To assess whether ALKBH5 plays an important role in SJP-associated autophagy regulation, ALKBH5 silencing and overexpression assays were performed in H/R-treated H9c2 cells after SJP administration. As demonstrated above, SJP inhibited autophagy and apoptosis triggered by ALKBH5 in H/R-treated cardiomyocytes. Subsequently, we confirmed that GSK3β/mTOR plays a crucial role in ALKBH5-dependent autophagy after SJP administration in H/R-treated H9c2 cells. These findings suggest SJP inhibits autophagy and apoptosis to protect H/R-treated H9c2 cells via ALKBH5/GSK3β/mTOR signaling, and these effects of SJP were also exerted by its two main constituents, including TMP and BOR.

In this study, we observed that BOR and SJP exerted comparable effects on autophagic activity and apoptosis in H/R-treated H9c2 cells, suggesting the main SJP constituents also efficiently regulate autophagy. In comparison with the TMP and BOR groups, SJP markedly decreased m6A amounts in cardiomyocytes, indicating multi-constituent herbal preparations may show higher effectiveness in regulating m6A compared with their active components considered individually. Moreover, BOR and SJP exerted comparable effects on ALKBH5 expression. Taken together, these results showed SJP affects autophagic activity mainly through BOR in H/R-induced H9c2 cells. However, BOR is often used in combination with other TCMs rather than alone to treat CVD due to its potential side effects. In the formulation of SJP, the dosage of BOR is within the safe range. In addition, BOR significantly relieved MIRI in this study, thereby providing an important basis for developing novel therapeutics for MIRI. The concept of “synergy” in TCM is based on synergistic effects exerted by multi-component herbal preparations, which may result in enhanced pharmacological activities. In TCM, multi-herb medicines are administered in CVD cases on the basis of such synergistic, multi-target interactive effects.

Nevertheless, the present study had some limitations. Firstly, an ALKBH5 knockout animal model is warranted to provide sufficient evidence for the notion that SJP inhibits autophagy and protects H/R-treated H9c2 cells via the ALKBH5/GSK3β/mTOR pathway. Additionally, SJP is a national patient formula in China, whose extraction process and extracted ingredients are not disclosed to the public; thus, we only considered TMP and BOR as active constituents and failed to assess other ingredients that might demonstrate superior therapeutic effects.

Extending the mechanism underpinning ALKBH5-related regulation of autophagy and apoptosis in H/R-treated cardiomyocytes may clarify the roles of involved effectors as possible therapeutic targets in MIRI and further determine the underlying mechanism of SJP in alleviating MIRI. Although an optimal method to prevent and treat MIRI is currently lacking, several studies have demonstrated that TCM improves MIRI. For example, Danshensu [34], Panax notoginseng [35], and Ligusticum chuanxiong [36] can alleviate MIRI, which may be related to the inhibition of excessive autophagy, suggesting that clinical application of natural products or TCM compounds may provide a new avenue for MIRI treatment.

## Conclusion

**Fig. 7:**
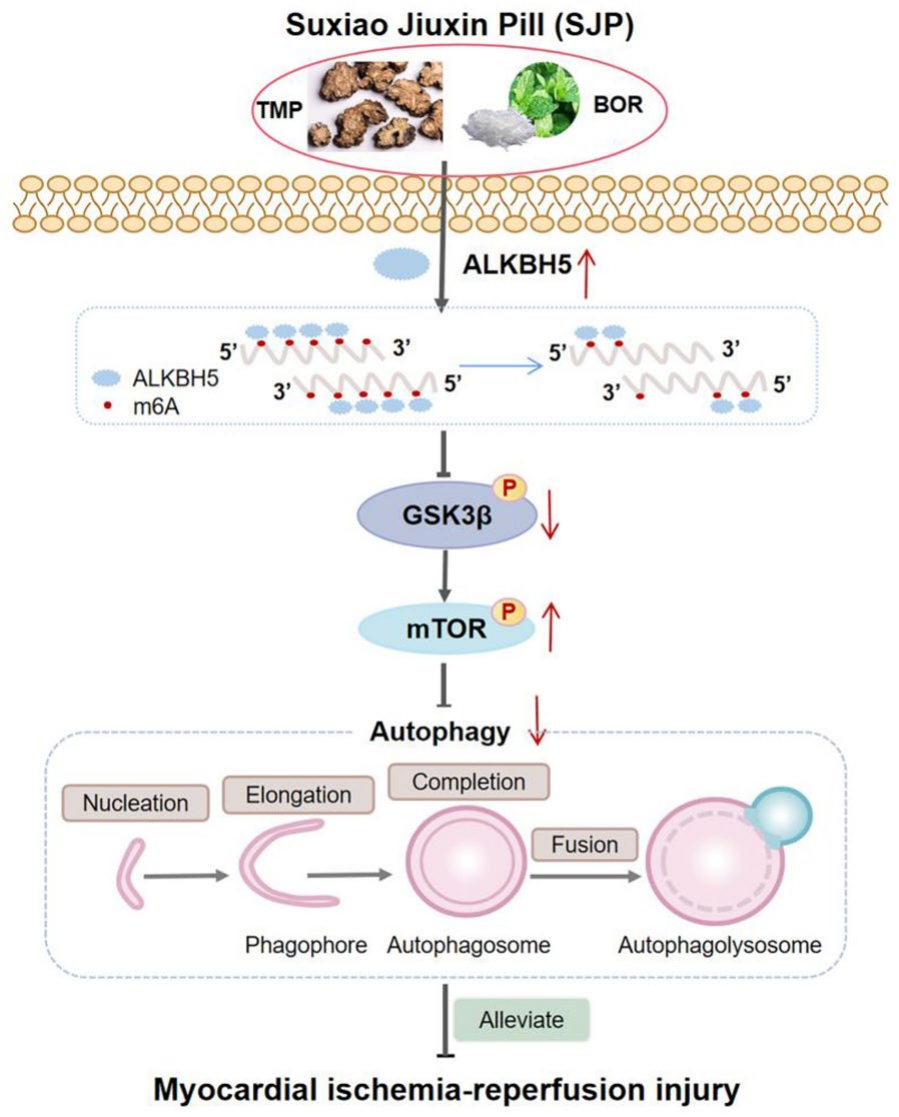
SJP administration improves MIRI in rats, through the ALKBH5/GSK3β/mTOR pathway, thanks to its two major ingredients, in H/R-treated H9c2 cells

In conclusion, SJP administration improves MIRI in rats, likely through the ALKBH5/GSK3β/mTOR pathway, involving the two major SJP components in H/R-induced H9c2 cells (Fig. 7).

## Supplementary Information

**Additional file 1**: **Fig S1.** National technical secret certificate of Suxiao Jiuxin Pills (SJP). **Fig S2.** HPLC fingerprint of Suxiao Jiuxin Pills. **Fig S3.** HPLC fingerprint of Ferulic acid. **Fig S4.** HPLC fingerprint of Senkyunolide I. **Fig S5.** HPLC fingerprint of Senkyunolide A. **Fig S6.** Establishment of hypoxia-reoxygenation model and determination of reoxygenation time. **Fig S7.** Determination of drug concentration. **Fig S8.** SJP and ALKBH5 overexpression inhibit excessive autophagy (*n*=3). *, #, *P *< 0.05 versus NC + H/R group. **Fig S9.** Virus interference efficiency of ALKBH5 and GSK3β by qPCR (*n*=3). **Table S1.** Specific situation of main components of SJP (1). **Table S2.** Primer information.

## Abbreviations

AMI: Acute myocardial infarction
MIRI: Myocardial ischemia—reperfusion injury
SJP: Suxiao jiuxin pills
AAR: Dangerous myocardial area
CVD: Cardiovascular disease
ACS: Acute coronary syndrome
LAD: Left anterior descending
ALKBH5: Human AlkB homolog H5
GSK3β: Glycogen synthase kinase 3 beta
mTOR: Mammalian target of rapamycin
m6A: N6 methyladenosine
TMP: Tetramethylpyrazine
BOR: Borneol
