# Supplementary material for: Suxiao Jiuxin Pill alleviates myocardial ischemia–reperfusion injury through the ALKBH5/GSK3β/mTOR pathway
Source: Chin Med. 2023 Mar 23;18:31. doi: 10.1186/s13020-023-00736-6 (PMC10037824; doi:10.1186/s13020-023-00736-6)
Supplement: Supplementary file 1 — Additional file 1: Fig S1. National technical secret certificate of Suxiao Jiuxin Pills (SJP). Fig S2. HPLC fingerprint of Suxiao Jiuxin Pills. Fig S3. HPLC fingerprint of Ferulic acid. Fig S4. HPLC fingerprint of Senkyunolide I. Fig S5. HPLC fingerprint of Senkyunolide A. Fig S6. Establishment of hypoxia-reoxygenation model and determination of reoxygenation time. Fig S7. Determination of drug concentration. Fig S8. SJP and ALKBH5 overexpression inhibit excessive autophagy (n=3). *, #, P < 0.05 versus NC + H/R group. Fig S9. Virus interference efficiency of ALKBH5 and GSK3β by qPCR (n=3). Table S1. Specific situation of main components of SJP (1). Table S2. Primer information. [file 13020_2023_736_MOESM1_ESM.docx]

Additional File 1 of Suxiao Jiuxin Pill alleviates myocardial ischemia-reperfusion injury through the ALKBH5/GSK3β/mTOR pathway

# Supplementary Figures and Tables

## *Supplementary Figures*

**National technical secret certificate of Suxiao Jiuxin Pills (SJP)**


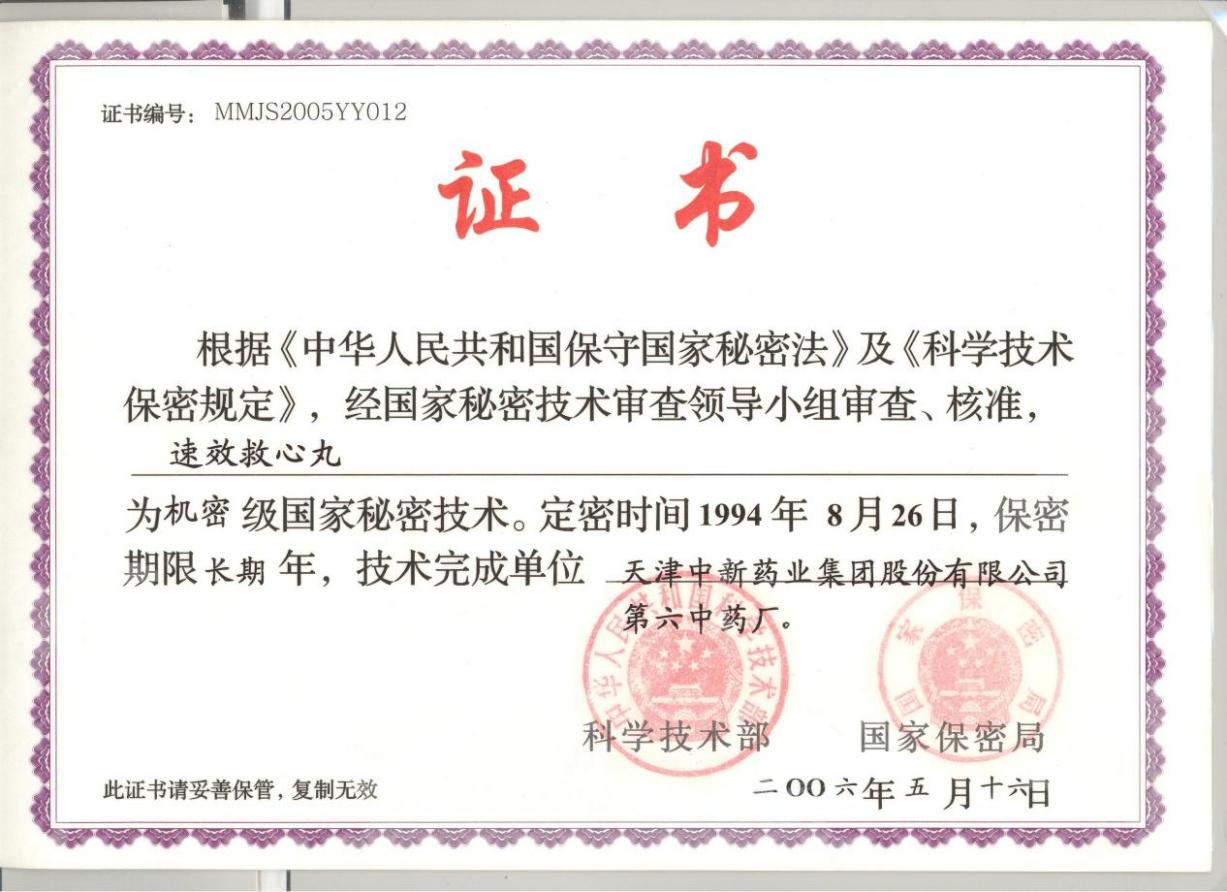
Suxiao Jiuxin Pill is a classified technology of state; therefore, full details on the ratio of each ingredient in the formulation, the method of extraction and the preparation of the final formulation are not clear (**Additional file 1: Fig S1**).

**Additional file 1: Fig S1.** National technical secret certificate of Suxiao Jiuxin Pills (SJP)

Translation: National technical secret certificate of Suxiao Jiuxin pills

Certificate No：MMJS2005YY012

According to the "Law of the People's Republic of China on Keeping Confidentiality of State Secrets" and "Regulations on Science and Technology Secrets", and after review and approval by the State Technology Secrets Review Leading Group, Suxiao Jiuxin Pills is a classified state secret technology. The confidentiality date was August 26, 1994, and the confidentiality period was long-term. The technical completion unit is the Zhongxin Pharmaceutical Group sixth TCM factory.

Ministry of Science and Technology of the People’s republic of China

National Administration of State Secrets Protection

**High-Performance Liquid Chromatography (HPLC) of SJP and its main active components**

**Additional file 1:
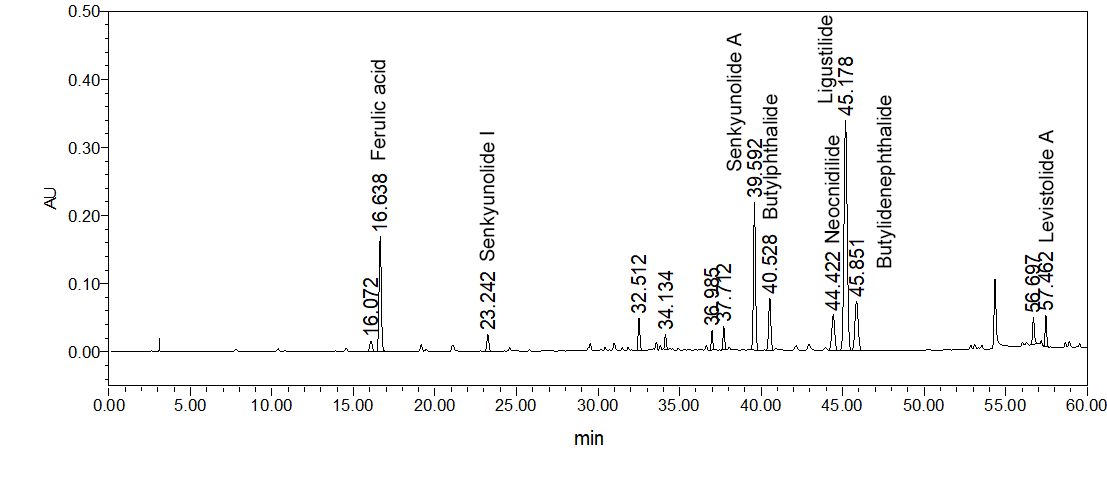
Fig S2.** HPLC fingerprint of Suxiao Jiuxin Pills

**Additional file 1:
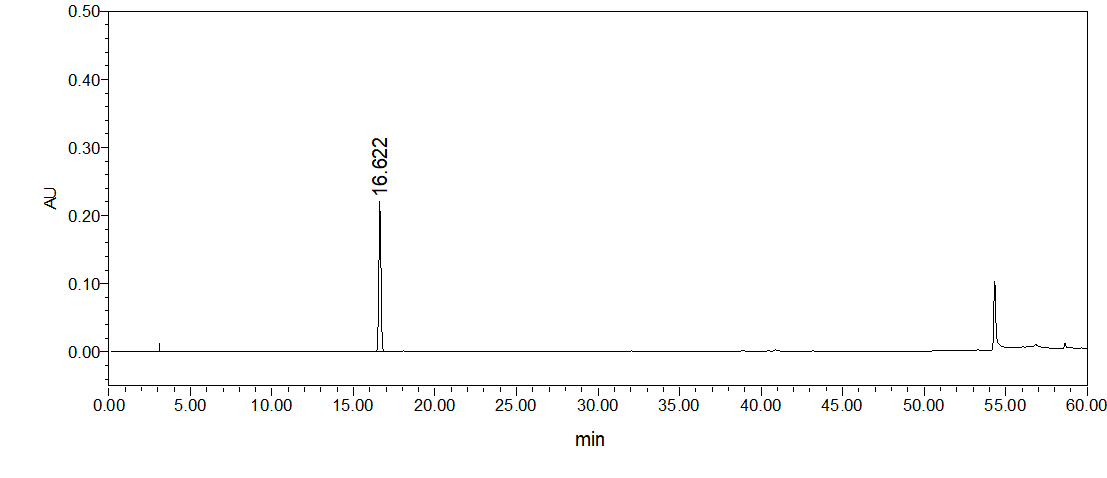
Fig S3.** HPLC fingerprint of Ferulic acid

**Additional file 1:**
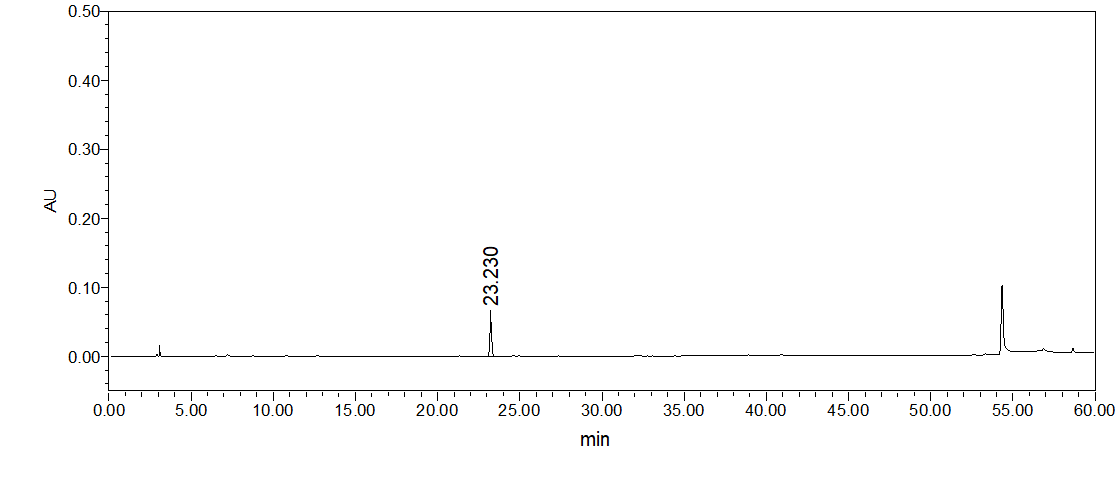
**Fig S4.** HPLC fingerprint of Senkyunolide I

**Additional file 1:**
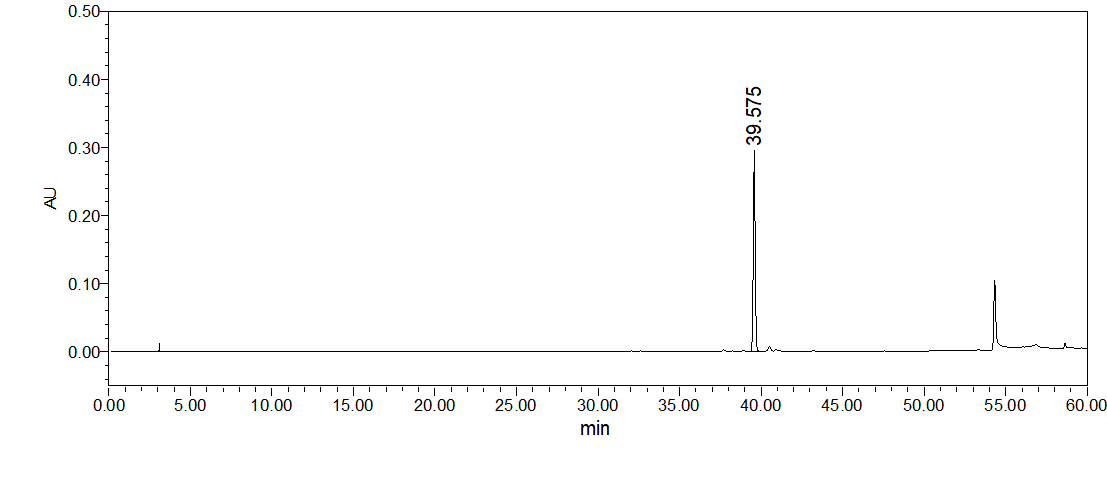
**Fig S5.** HPLC fingerprint of Senkyunolide A

**Establishment of hypoxia-reoxygenation model and determination of reoxygenation time**

H9c2 cells were cultured in high glucose DMEM with 10% fetal bovine serum (FBS), and subcultured in a 5% CO_2_ incubator for 3 passages to establish a hypoxia/reoxygenation (H/R) model. The medium was replaced with low-glucose and serum-free DMEM, cultured for 2 h under hypoxic conditions (1% O_2_, 94% N_2_, 5% CO_2_) and then reoxygenated (95% air, 5% CO_2_) for 0 h, 2 h, 4 h, 6 h, respectively. The results showed that the cell viability of normoxic groups was superior to different reoxygenation time groups. Reoxygenation for 4 h could restore cell viability. Therefore, the subsequent hypoxia and reoxygenation models all adopted hypoxia for 2 h and reoxygenation for 4 h (**Additional file 1: Fig S6**).

**Additional file 1:**
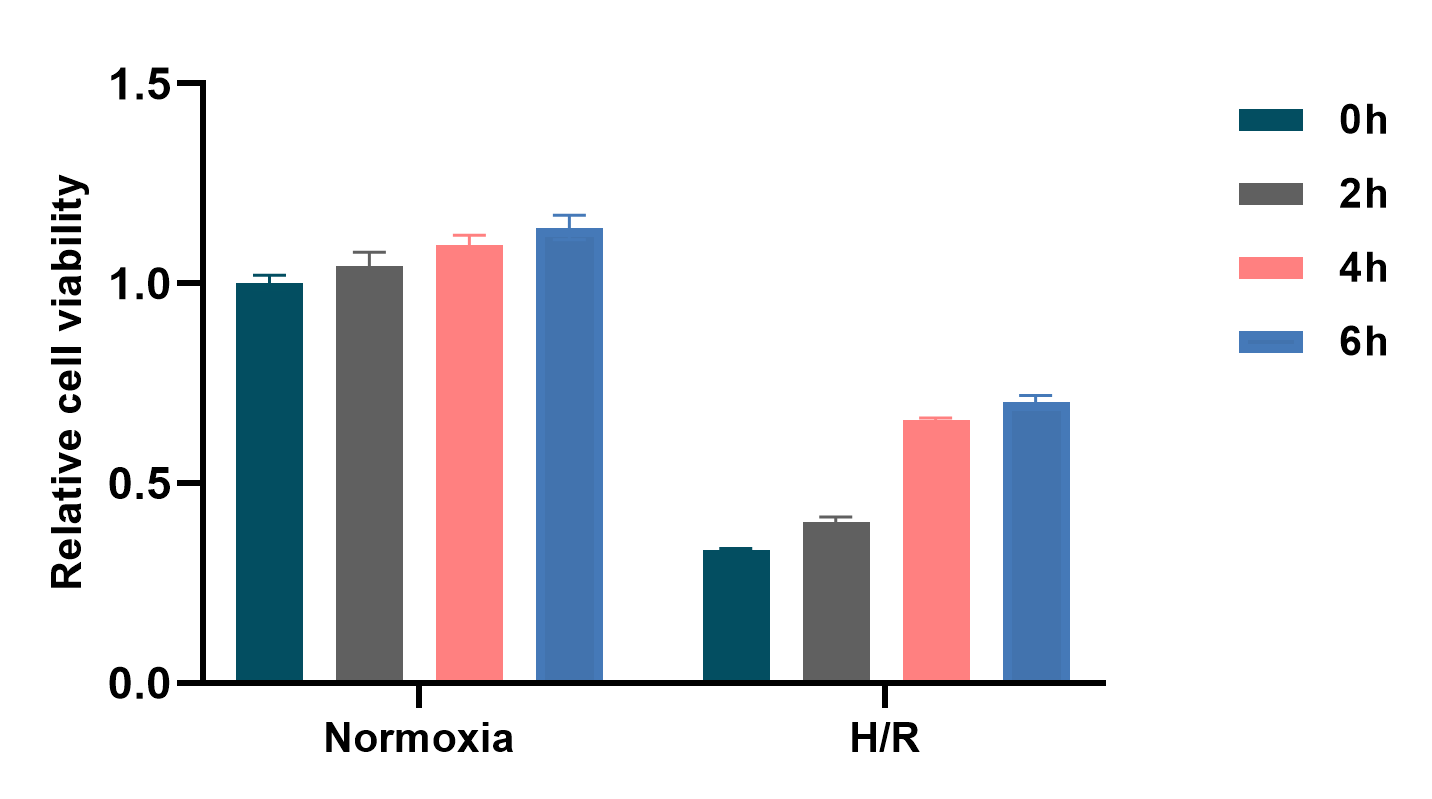
**Fig S6.** Establishment of hypoxia-reoxygenation (H/R) model and determination of reoxygenation time.

**Determination of drug concentration**

## Suxiao Jiuxin Pill (SJP) and its active ingredients ligustrazine (TMP) and borneol (BOR) were diluted and dissolved to 50 mg/ml, 100 mg/ml, and 25 mg/ml with 0.5% ethanol. CHIR99021 and rapamycin were diluted and dissolved to 0.01uM, 0.1uM, and 1uM. The results showed that under the condition of hypoxia/reoxygenation (H/R), SJP, TMP, BOR, and CHIR99021 all restored cell viability. The optimal concentration was 50ug/mL, 100ug/ mL, 25ug/ mL, and 1uM, respectively. Rapamycin inhibited cell
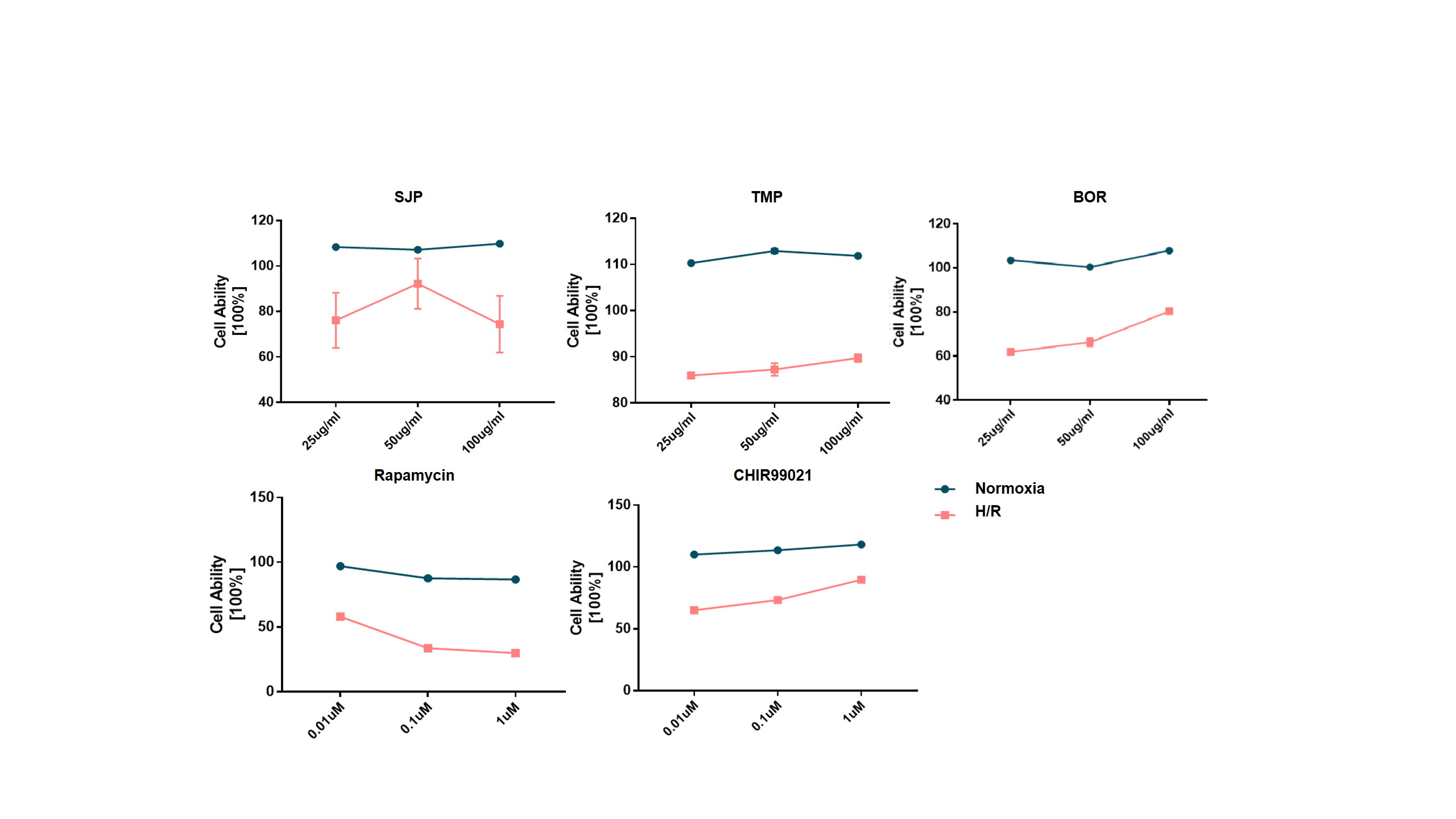
viability under H/R, and the optimal concentration was 0.1uM. There was little effect on cell viability under normoxic conditions (**Additional file 1:** Fig S7).

**Additional file 1: Fig S7.** Determination of drug (SJP, TMP, BOR, Rapamycin, and CHIR99021) concentration by CCK8 assay.

**SJP and ALKBH5 overexpression inhibit excessive autophagy**

SJP and ALKBH5 overexpression inhibit excessive autophagy rat ALKBH5 overexpressed lentivirus (F-ACCACCAAACGGAAGTACCA; R-CGTTGTACAGGCCCTTCTCA) was infected with H9c2 cells, negative lentivirus infection control group and blank cell group were set, then cells were collected after 48h later and the overexpression effect was detected by q-PCR (**Additional file 1: Table S2**). Consistent with upregulation of *ALKBH5, GSK3β* mRNA and autophagy-related genes were dramatically downregulated and *mTOR* mRNA was upregulated in H/R-induced cardiomyocytes with SJP pretreatment (*P* <0.05) (**Additional file 1: Fig S8**).

**Additional file 1:**
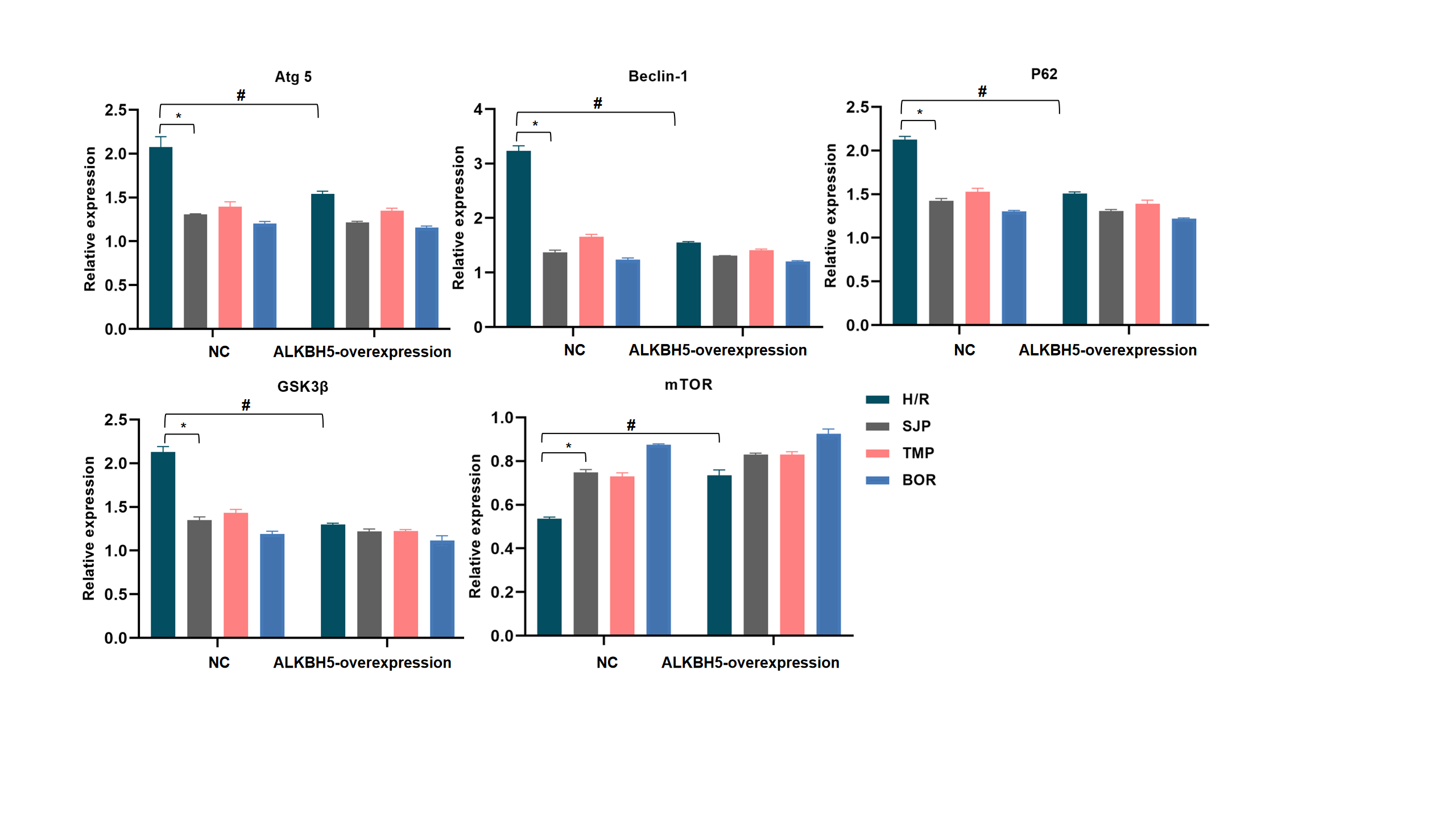
**Fig S8.** SJP and ALKBH5 overexpression inhibit excessive autophagy (*n*=3). *, #, *P* < 0.05 versus NC + H/R group.

**Virus interference efficiency of ALKBH5 and GSK3β**

**
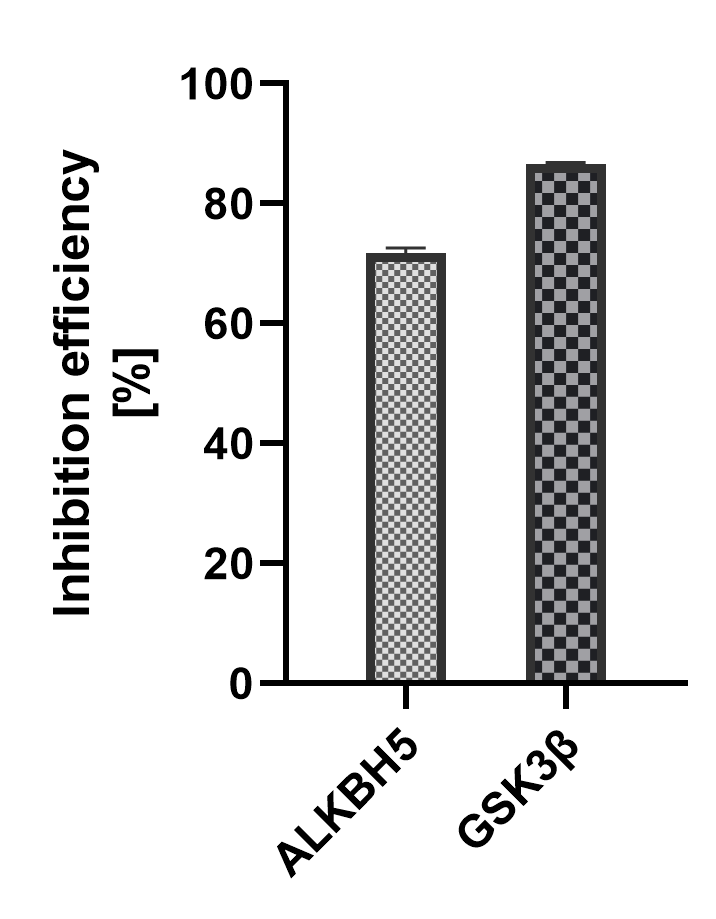
**Rat ALKBH5 shRNA lentivirus and GSK3β shRNA lentivirus were infected with H9c2 cells, negative lentivirus infection control group and blank cell group were set, then cells were collected after 48h later and the inhibition effect was detected by q-PCR (**Additional file 1: Fig S9**).

**Additional file 1: Fig S9.** Virus interference efficiency of ALKBH5 and GSK3β by qPCR (*n*=3).

## *Supplementary Tables*

**Specific situation of main components of SJP**

SJP is composed of Rhizome *Ligusticum Chuanxiong* (Chuanxiong, *Ligusticum chuanxiong Hort.*) and *Dryobalanops aromatica* C.F.Gaertn. (Bingpian). The specific situation of main components of SJP is presented as follows (**Additional file 1: Table S1**).

**Additional file 1: Table S1.** Specific situation of main components of SJP (1)

| Main components | Botanical species | Plant parts | Main active components |
| --- | --- | --- | --- |
| Chuanxiong | Rhizome  *Ligusticum*  *Chuanxiong* | The dried  root | Ferulic acid, Senkyunolide A-I, Butylphthalide, Neocnidilide, Butylidenephthalide, Ligustilide, Levistolide A, Borneolum Syntheticum, Tetramethylpyrazine, Senkyunolide M or Q, Senkyunolide N, E-ligustilide, Z-ligustilide, Ligustilide dimmer, Angelicide, Levistolide A, Butylphthalide, Butylidenephthalide, 4,7-Dihydroxy-3-butylphthalide, 3- Butyl-4-hydroxyphthalide, Feruloylquinic acid, Vanillic acid, Caffeic acid, Caffeoylquinic acid, Dicaffeoylquinic acid, Cnidilide |
| Bingpian | *Cinnamomum*  *camphora (L.)*  Presl | The fresh  branches  and leaves |  |

**Table S2.** Primer information

**Primer sequence information**

Primer sequence information of *ALKBH5* mRNA, *GSK3β* mRNA, *mTOR* mRNA, *Atg-5* mRNA, *Beclin-1* mRNA, and *p62* mRNA in ALKBH5 overexpression experiments.

**Additional file 1: Table S2.** Primer sequence information in ALKBH5 overexpression experiments.

| **Primer** | **Sequence** |
| --- | --- |
| ALKBH5 F | 5’-GACCTGCGTGAGAAGCTCAA-3’ |
| ALKBH5 R | 5’-TGGTACTTCCGTTTGGTGGTC-3’ |
| GSK3β F | 5’-TGGACAGACCAATAACGCCG-3’ |
| GSK3β R | 5’-GTGACCAGTGTTGCTGAGTG-3’ |
| mTOR F | 5’-AATCGTGGTGGCTCTTGGAG-3’ |
| mTOR R | 5’-GGAGGCAACAACAAGTGCAG-3’ |
| ATG 5 F | 5’-ACGTGTGGTTTGGACGGATT-3’ |
| ATG 5 R | 5’-TGTTCCAAGGCAGAGCTGAG-3’ |
| Beclin1 F | 5’-CCCAGCCAGGATGATGTCTAC-3’ |
| Beclin1 R | 5’-AGTCTCCGGCTGAGGTTCTC-3’ |
| P62 F | 5'-CTGAGAAGGACTCGCTCGAC-3' |
| P62 R | 5'-TCAGTACCCGCTCTTTCAGC-3' |
| Actin F | 5'-ACACCCCAGCCATGTACGT-3 |
| Actin R | 5'-ATGGGCACAGTGTGGGTGA-3' |

# Reference

1. Qiang T, Li Y, Wang K, Lin W, Niu Z, Wang D, et al. Evaluation of potential herb-drug interactions based on the effect of Suxiao Jiuxin Pill on CYP450 enzymes and transporters. J Ethnopharmacol. 2021;280:114408.
